# Experimental Study on the Mechanical Properties and Water Stability of Loess Stabilized with Calcium Carbide Residue and Phosphogypsum

**DOI:** 10.3390/ma19214663

**Published:** 2026-11-02

**Authors:** Qizhi Hu, Xuan Peng, Qiang Ma, Gaoliang Tao

**Affiliations:** 1 School of Civil Engineering, Architecture and Environment, Hubei University of Technology, No. 28 Nanli Road, Hongshan District, Wuhan 430068, China; hqz0716@163.com (Q.H.); maqiang927@163.com (Q.M.); tgl1979@126.com (G.T.); 2 Hubei Provincial Bridge Safety Monitoring Technology and Equipment Engineering Technology Research Center, No. 28 Nanli Road, Hongshan District, Wuhan 430068, China

**Keywords:** calcium carbide residue, phosphogypsum, loess stabilization, unconfined compressive strength, water stability

## Abstract

Loess is susceptible to disintegration and strength loss upon water exposure, which may compromise the stability of loess subgrades. Conventional stabilization methods using cement or lime are relatively costly and involve substantial carbon emissions. To investigate the potential use of industrial solid wastes for loess stabilization, this study examined the effects of calcium carbide residue (CCR) and phosphogypsum (PG) on the unconfined compressive strength (UCS) and water stability of loess. Sixteen mixtures with different CCR and PG contents were prepared and tested. The results showed that the combined addition of CCR and PG improved both the strength and water stability of loess, indicating a positive combined effect between the two additives. The highest UCS of 520 kPa was obtained with 4% CCR and 18% PG, and the corresponding water stability coefficient was 84.1%. Further X-ray diffraction (XRD) and scanning electron microscopy (SEM) analyses suggest that the combined addition of CCR and PG promotes the formation of calcium silicate hydrate (C-S-H), calcium aluminosilicate hydrate (C-A-S-H), and needle-like ettringite (AFt) crystals. These reaction products fill the pores and bind the soil particles together, resulting in a denser and more coherent soil structure. The findings provide a reference for the utilization of industrial solid wastes in loess stabilization.

## 1. Introduction

Loess is characterized by high porosity, a loose structure, and low strength, making it susceptible to substantial strength loss upon wetting and limiting its direct use as subgrade fill [1]. Methods for loess stabilization include physical, biological, and chemical approaches. Biological stabilization commonly involves microbially induced carbonate precipitation (MICP). In this process, urease produced by microorganisms catalyzes the hydrolysis of urea, leading to the formation of carbonate ions (CO_3_^2−^), which react with calcium ions (Ca^2+^) in the cementation solution to precipitate calcium carbonate (CaCO_3_). The precipitated CaCO_3_ fills soil pores and binds soil particles together, thereby improving the engineering properties of loess [2,3]. Chemical stabilization involves the addition of binders such as lime and cement, which react with soil constituents to form cementitious products that improve the engineering properties of loess [4,5].

Conventional soil stabilization relies primarily on chemical binders such as lime and cement. However, their extensive use increases costs and contributes to carbon emissions, raising concerns about the sustainability of conventional stabilization methods [6,7]. Previous studies have shown that industrial solid wastes, including calcium carbide residue (CCR), slag, and phosphogypsum (PG), can be used to improve the engineering properties of loess [6,8,9]. CCR is a strongly alkaline by-product of acetylene production, consisting primarily of calcium hydroxide [Ca(OH)_2_], and can be used as an alkaline activator [7,10]. PG is a by-product of wet-process phosphoric acid production and consists mainly of calcium sulfate dihydrate (CaSO_4_·2H_2_O), with SiO_2_ present as an impurity [11]. In an alkaline environment, PG can provide sulfate ions that participate in hydration reactions and contribute to the formation of cementitious products [7]. The combined use of CCR and PG for loess stabilization is intended to integrate alkaline activation with sulfate activation. The addition of CCR and PG may promote reactions that produce ettringite (AFt) and calcium aluminosilicate hydrate (C-A-S-H). These reaction products can fill soil pores and bind soil particles together, thereby facilitating the beneficial utilization of industrial solid wastes [7,12,13].

This study investigates the stabilization of loess using CCR and PG. The effects of different CCR and PG contents on the unconfined compressive strength (UCS) and water stability of loess are evaluated to identify a suitable mixture proportion. Scanning electron microscopy (SEM) and X-ray diffraction (XRD) are further employed to characterize the microstructure of the stabilized loess and investigate the possible mechanisms underlying the observed changes in its engineering properties. The findings provide experimental evidence for the potential use of CCR and PG in loess stabilization for subgrade applications.

## 2. Experimental Program

### 2.1. Test Materials

**Figure 1:**
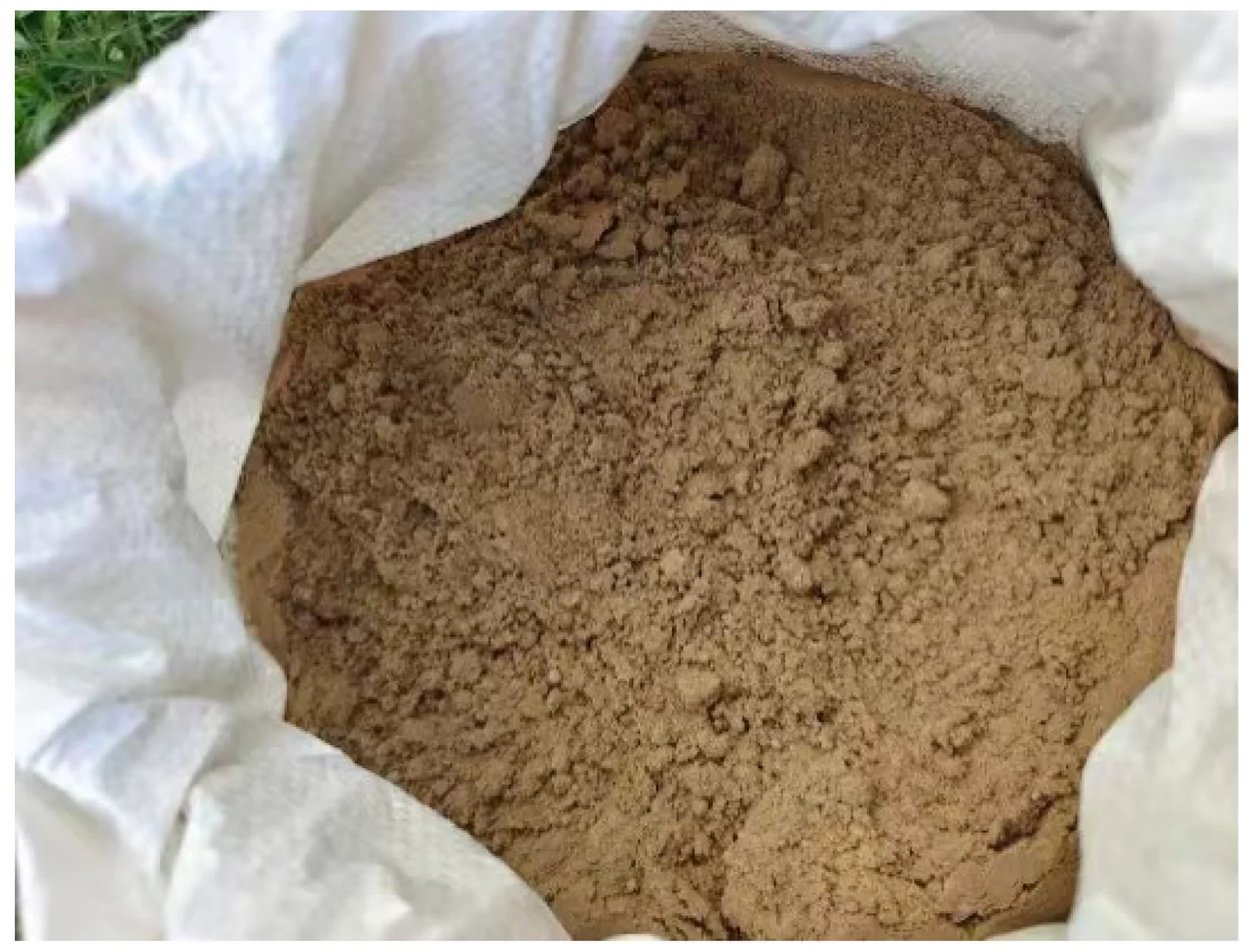
Experimental loess.

**Figure 2:**
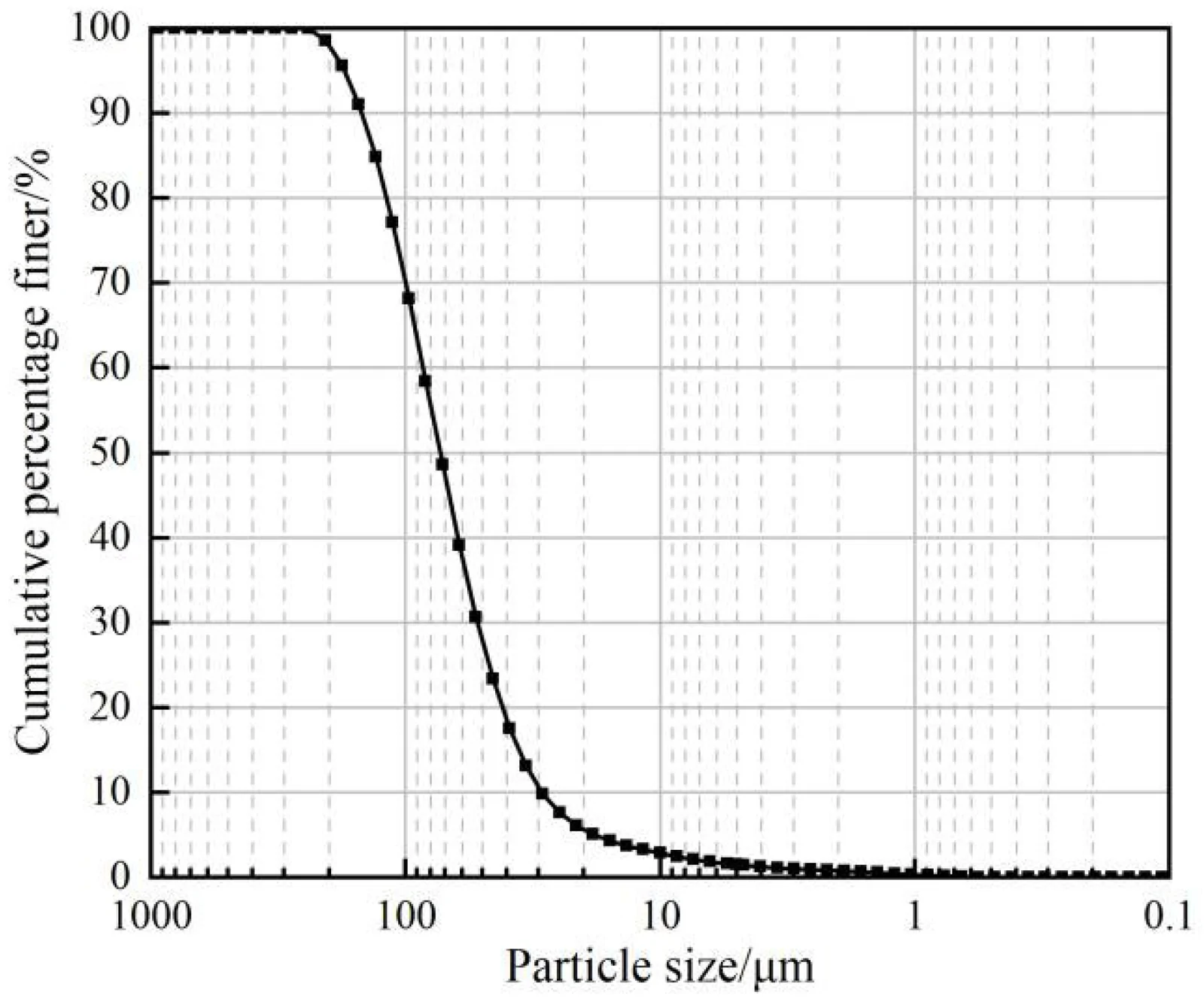
Particle-size distribution of loess.

Loess samples were collected from a campus construction site in Hubei Province, China, at a depth of approximately 2.5 m below the ground surface, as shown in Figure 1. The collected soil was yellowish-brown and exhibited a stiff-plastic consistency. Compaction and Atterberg limit tests were conducted in accordance with the Standard for Geotechnical Testing Method (GB/T 50123-2019) [14], and the resulting physical properties are presented in Table 1. The particle-size distribution was determined through particle-size analysis, as shown in Figure 2. The loess had an average particle size of approximately 60 μm and consisted predominantly of silt, with a relatively low clay content. These characteristics indicate that the soil may be unsuitable for direct use as subgrade fill.

The CCR used in this study was supplied by a materials company in Henan Province, China. The supplier was Henan Dingnuo Purification Materials Co., Ltd. (Zhengzhou, China).

**Figure 3:**
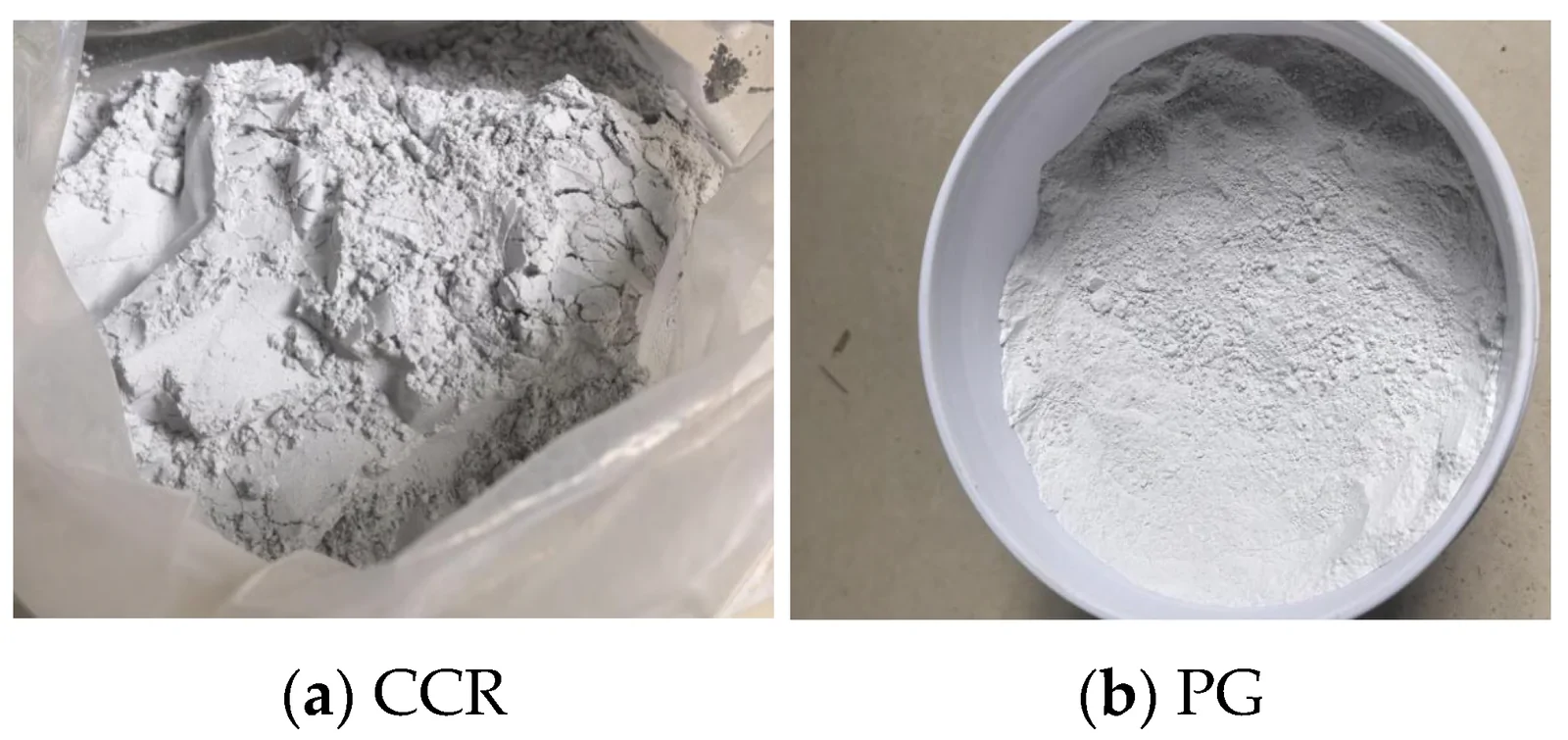
Photographs of CCR and PG used in this study.

**Figure 4:**
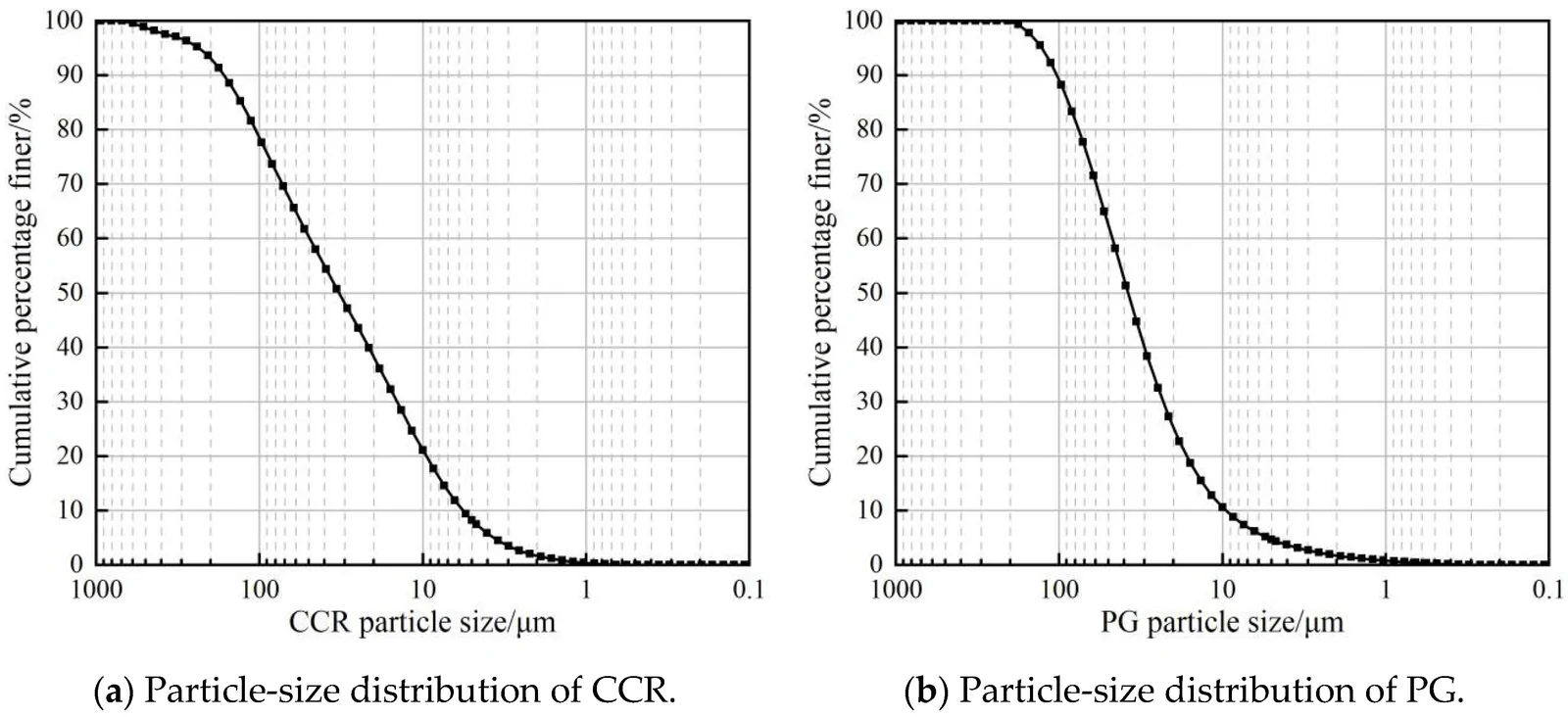
Particle gradation curve of calcium carbide slag and phosphogypsum.

As shown in Figure 3a, CCR is a grayish-white powder with a slight odor, a density of 2.253 g/cm^3^, and an average particle size of 45.2 μm. Its particle-size distribution and chemical composition are presented in Figure 4a and Table 2, respectively. The main chemical components of CCR, expressed as oxides, are CaO, SiO_2_, and Al_2_O_3_, with CaO accounting for 79.89%.

The PG used in this study was obtained from a chemical plant in Hubei Province, China, The supplier was Hubei Xiangyun (Group) Chemical Industry Co., Ltd. (Huanggang, China) and consisted mainly of CaSO_4_·2H_2_O. As shown in Figure 3b, PG is a grayish-white powder with a density of 2.219 g/cm^3^ and a relatively large specific surface area. Its average particle size is 21.8 μm, while its particle-size distribution and chemical composition are presented in Figure 4b and Table 3, respectively.

### 2.2. Specimen Preparation and Testing Procedures

Considering the need to ensure sufficient hydration reactions while avoiding an excessive SO_4_^2−^ content in the soil, the total CCR and PG content was limited to 30% in this study. Four CCR contents of 0%, 4%, 8%, and 12% and four PG contents of 0%, 6%, 12%, and 18% were selected. The four CCR contents were combined with the four PG contents, resulting in a total of 16 mixtures, as listed in Table 4. The contents of CCR and PG were calculated as percentages of the dry mass of loess. Sample preparation was conducted in accordance with the Standard for Geotechnical Testing Method (GB/T 50123-2019) [14]. The field-collected loess was air-dried, crushed, and passed through a 2 mm sieve. The required amounts of loess, CCR, and PG were weighed according to the designed proportions and thoroughly mixed. A constant water content of 18%, corresponding to the optimum moisture content of the untreated loess, was adopted for all mixtures to ensure consistent specimen-preparation conditions. The mixtures were then sealed and allowed to equilibrate for 24 h before specimen preparation. The prepared mixture was compacted in three layers in a three-piece split mold with a diameter of 39.1 mm and a height of 80 mm. Each layer was compacted with 25 blows, and the surface was lightly scarified before placing the next layer. After compaction, the specimens were extruded from the mold, sealed in plastic bags, and cured in a standard curing chamber at (22 ± 2)°C and a relative humidity of at least 95%. This study primarily investigates the effects of different CCR and PG contents on the strength and water stability of loess. Curing age was not considered as an experimental variable. To eliminate the influence of curing-age differences on comparisons among specimens with different mixture proportions and to allow sufficient time for the formation of the main reaction products, all specimens were cured for 28 d.

UCS tests were conducted in accordance with the Standard for Geotechnical Testing Method (GB/T 50123-2019) [14]. Three replicate specimens were prepared for each mixture. After standard curing for 28 d, the specimens were tested using a WHY-200 microcomputer-controlled electronic universal testing machine (Shanghai Hualong Test Instruments Co., Ltd., Shanghai, China) at a loading rate of 1.00 mm/min. Loading was continued until the axial strain reached 8%, at which point the test was terminated. The mean UCS and standard deviation (SD) were calculated from the three replicate specimens for each mixture, and the mean UCS was used as the representative value. The variability of the UCS results is presented as mean ± SD (*n* = 3).

The water stability test was conducted in accordance with Soil Stabilizing Admixtures (CJ/T 486-2015) [15]. The water stability of the specimens was evaluated using the water stability coefficient (WSC) [16,17]. The standard-cured UCS used for calculating the WSC was taken directly from the UCS test results described above ($q_{u}$); therefore, no additional standard-cured specimens were prepared. For each mixture, three replicate specimens were separately prepared for the water-immersion test. These specimens were subjected to standard curing for 27 d, followed by water immersion for 1 d, resulting in a total test period of 28 d, consistent with that of the standard-cured specimens. After immersion, UCS tests were conducted, and the mean UCS of the three replicate specimens was taken as the post-immersion UCS ($q_{\mathrm{us}}$). The WSC was calculated using Equation (1).

(1) $$r=\frac{q_{\mathrm{us}}}{q_{u}}\times 100\%$$

In the equation: *r* is the water stability coefficient (WSC); $q_{\mathrm{us}}$ is the UCS of the specimen after 27 d of standard curing followed by 1 d of water immersion, in kPa; and ($q_{u}$) is the UCS of the specimen after 28 d of standard curing, in kPa.

To investigate the microstructural and mineralogical mechanisms underlying the stabilization of loess by CCR and PG, specimens from four representative groups, including untreated loess, CCR-stabilized loess, PG-stabilized loess, and CCR-PG-stabilized loess, were selected for SEM and XRD analyses [8,9]. For SEM analysis, representative fragments of approximately 1 cm^3^ were cut from the specimens, dried, sputter-coated with gold, and examined by SEM (Carl Zeiss Microscopy GmbH, Oberkochen, Germany). For XRD analysis, diffraction patterns were collected using a D8 Advance X-ray diffractometer (Bruker AXS GmbH, Karlsruhe, Germany) over a 2θ range of 5–90°, at a scanning rate of 10°/min and a step size of 0.02°.

## 3. Results and Discussion

### 3.1. Stress–Strain Characteristics of Stabilized Loess

**Figure 5:**
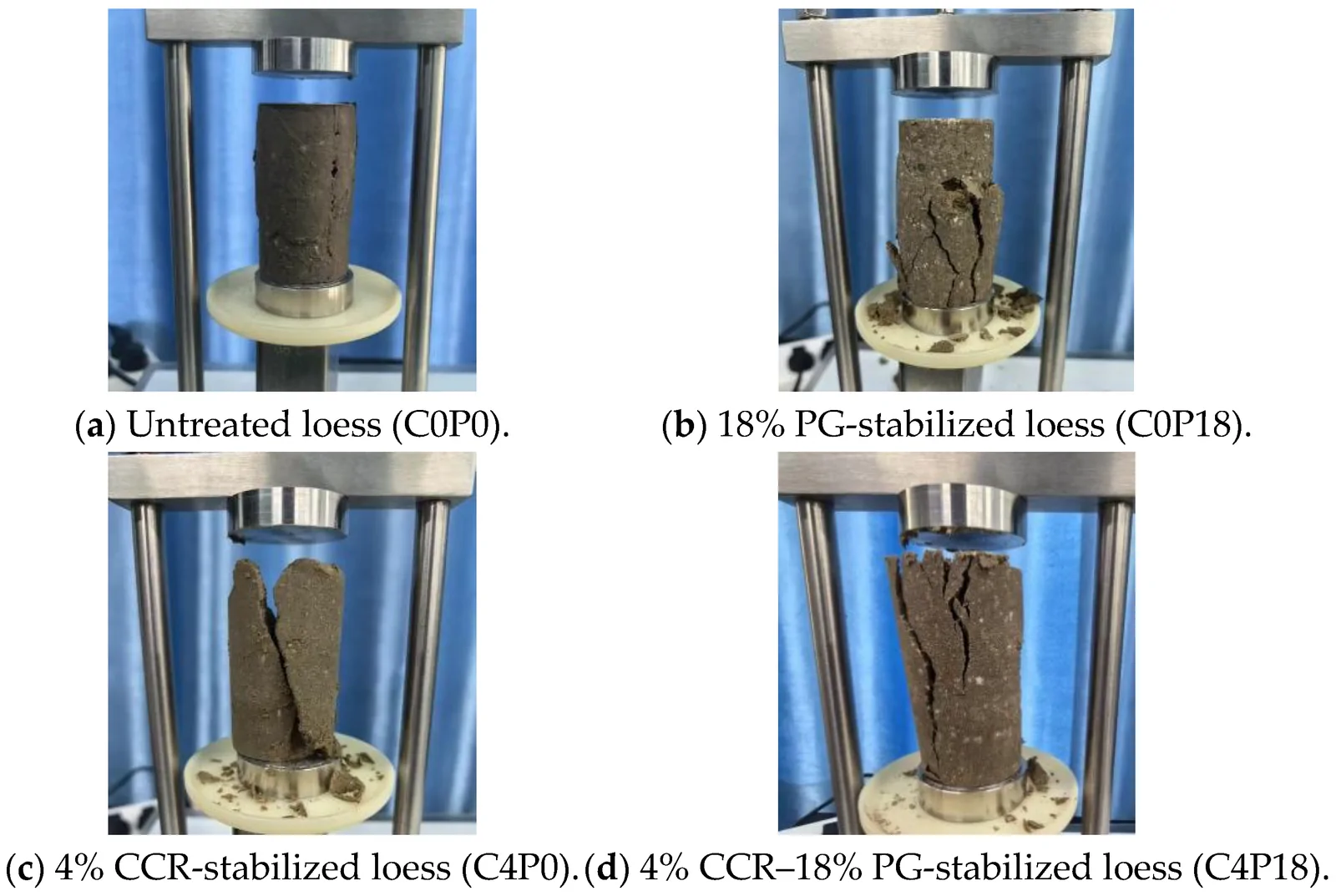
Failure patterns of untreated and selected stabilized loess specimens.

Figure 5 shows the typical failure patterns of the stabilized loess specimens. As shown in Figure 5a, the untreated loess specimen exhibited pronounced lateral bulging and axial shortening under compression, without the development of obvious cracks. The specimen gradually lost its integrity, indicating a predominantly ductile deformation mode. As shown in Figure 5b, the specimen containing 18% PG exhibited several fine cracks at failure; however, its overall integrity remained relatively poor, indicating that the improvement provided by PG alone was limited. As shown in Figure 5c, the specimen stabilized with CCR alone exhibited a more pronounced brittle response. Diagonal cracks developed through the cylindrical specimen, accompanied by local spalling near the edges, while the specimen largely retained its overall cylindrical shape. In contrast, the specimen containing 4% CCR and 18% PG exhibited a distinct axial splitting failure, as shown in Figure 5d. Nearly vertical through-cracks developed along the specimen, with relatively smooth and well-defined fracture surfaces. Compared with untreated loess, the CCR–PG-stabilized specimen exhibited a transition from ductile deformation to a more brittle failure mode while maintaining greater overall integrity. This behavior is consistent with enhanced interparticle cementation and a denser soil structure induced by the formation of cementitious reaction products [8,18].

**Figure 6:**
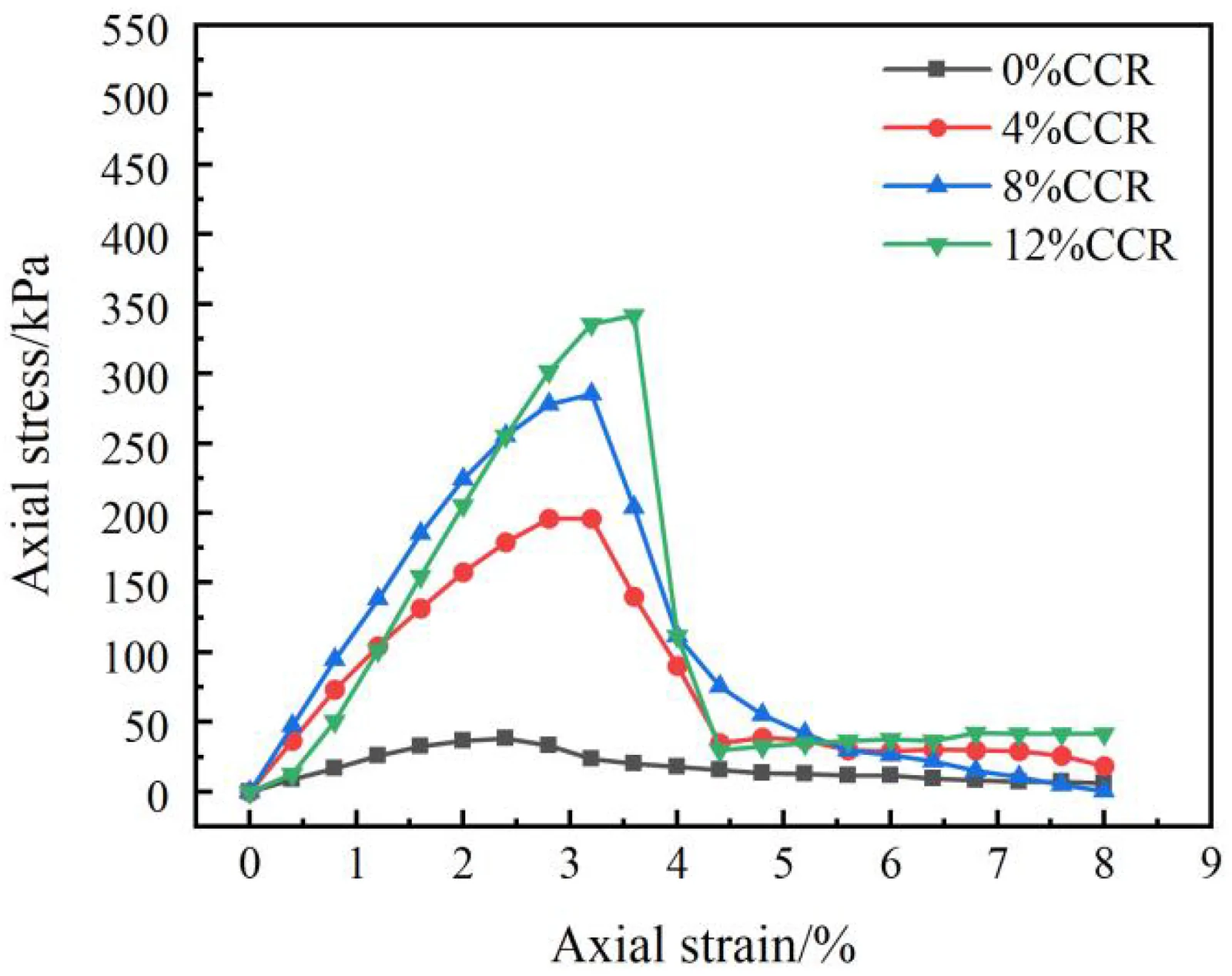
Stress–strain curves of stabilized loess with different CCR contents at 0% PG.

**Figure 7:**
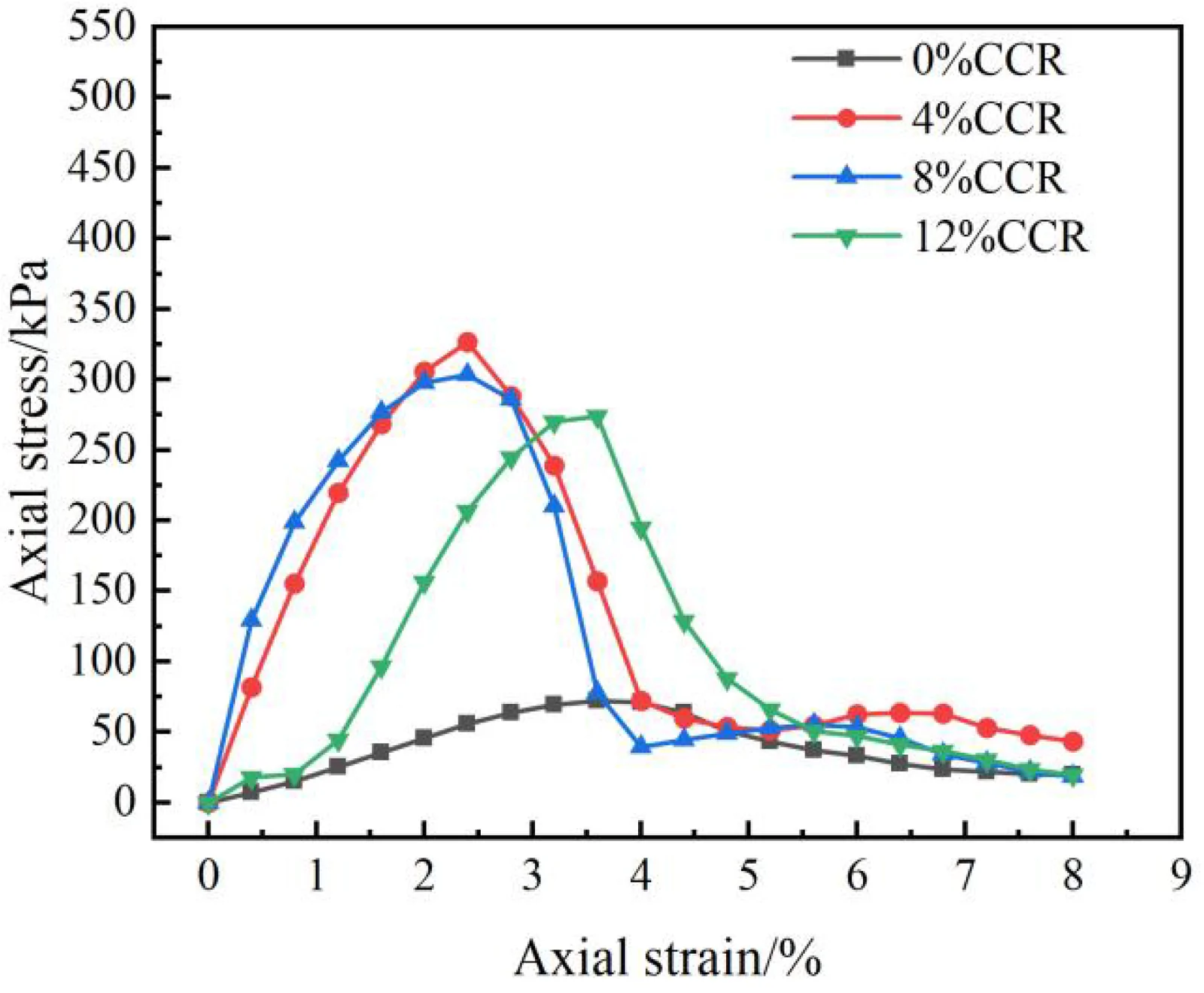
Stress–strain curves of stabilized loess with different CCR contents at 6% PG.

**Figure 8:**
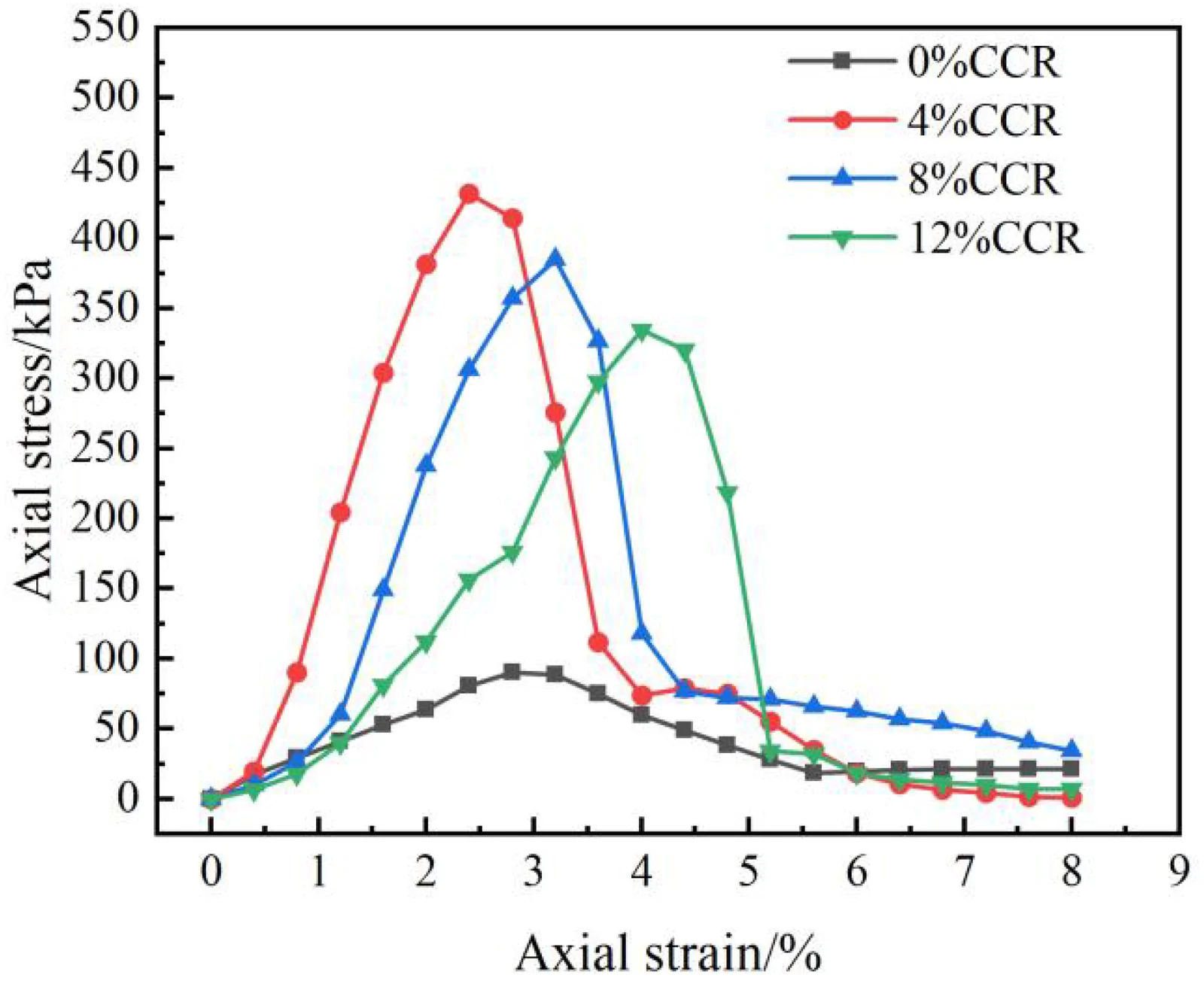
Stress–strain curves of stabilized loess with different CCR contents at 12% PG.

**Figure 9:**
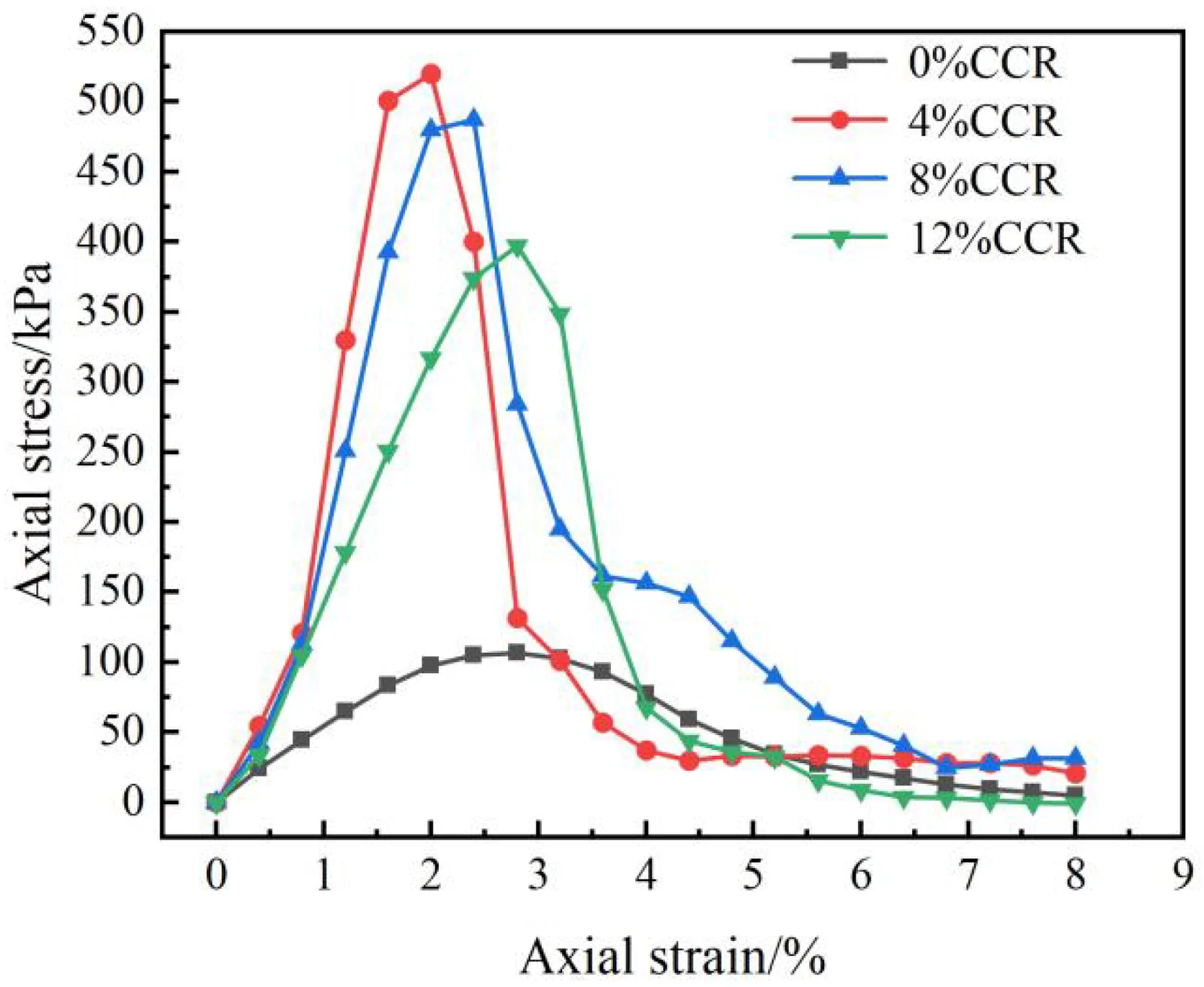
Stress–strain curves of stabilized loess with different CCR contents at 18% PG.

Figure 6, Figure 7, Figure 8 and Figure 9 show the stress–strain curves obtained from the UCS tests at different CCR and PG contents. Under axial compression, the stress–strain curves of the stabilized loess generally exhibit three stages: elastic deformation, plastic deformation, and failure, with the stress first increasing and then decreasing with increasing axial strain. As shown in Figure 6, when the PG content was 0%, i.e., when CCR was added alone, the UCS gradually increased with increasing CCR content, accompanied by a gradual increase in peak strain. At a CCR content of 12%, the UCS and peak strain reached their maximum values of 342.2 kPa and 3.8%, respectively.

As shown in Figure 7, Figure 8 and Figure 9, at PG contents of 6%, 12%, and 18%, the post-peak stress drop of the stabilized loess became more pronounced with increasing CCR content, and the failure behavior gradually shifted from ductile deformation to brittle failure. The peak strain first decreased and then increased with increasing CCR content, whereas the UCS first increased and then decreased. Taking Figure 7 as an example, when the CCR content increased from 0% to 4%, the peak strain decreased from 3.6% to 2.4%, while the UCS increased from 72.2 kPa to 326.7 kPa. However, when the CCR content increased further from 4% to 12%, the peak strain increased from 2.4% to 3.7%, whereas the UCS decreased from 326.7 kPa to 274.3 kPa. This behavior may be attributed to the fact that an appropriate CCR content provides a favorable alkaline environment and calcium source, thereby promoting reactions involving PG and reactive components in the soil and increasing the formation of cementitious products. These products enhance interparticle bonding and contribute to the formation of a denser and more brittle cemented structure. In contrast, excessive CCR may disturb the reaction balance and reduce the efficiency of cementitious product formation, leading to a decrease in UCS and an increase in peak strain [7,19].

### 3.2. Unconfined Compressive Strength of Stabilized Loess

**Figure 10:**
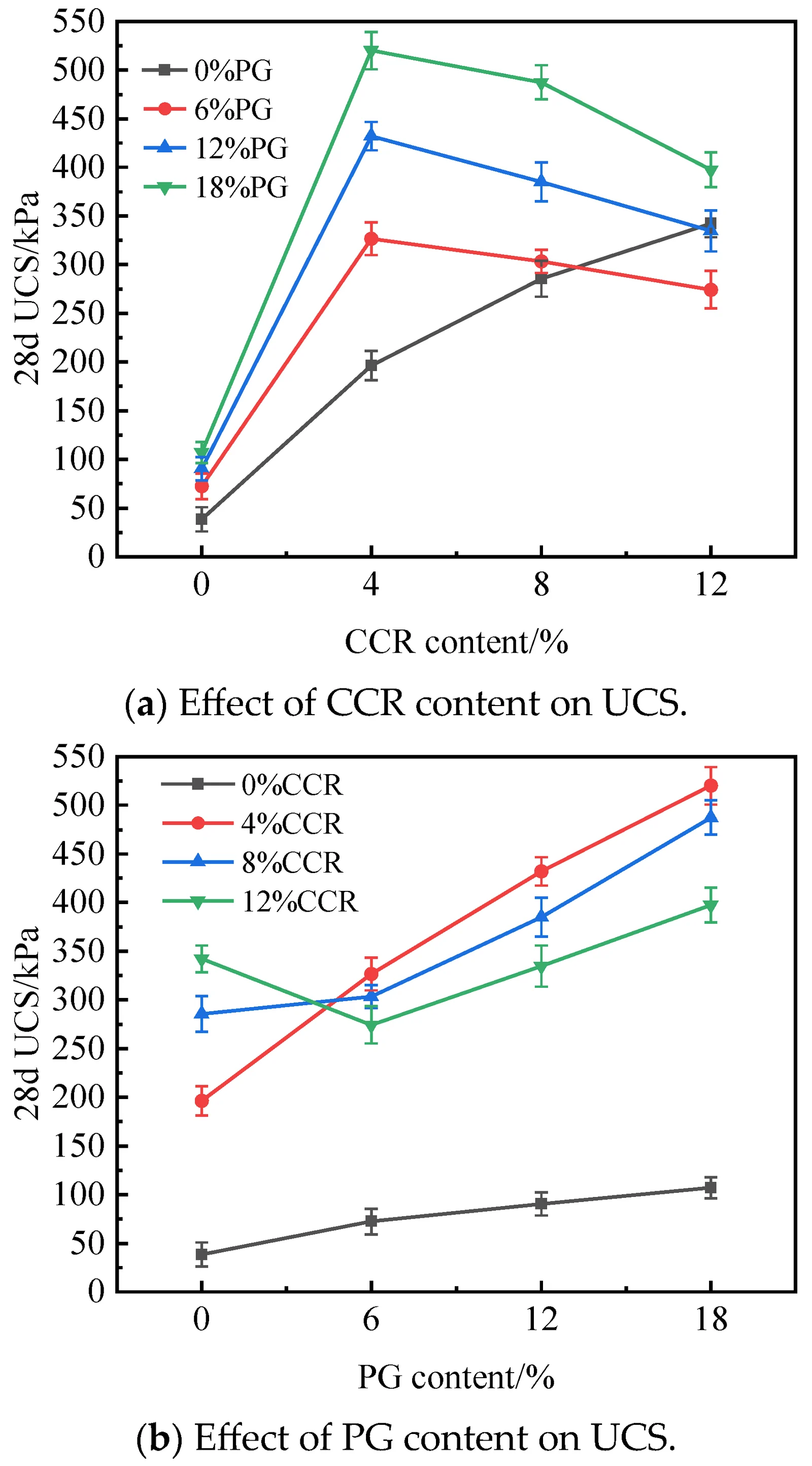
Effect of stabilizer content on UCS.

Figure 10 shows the relationship between stabilizer content and the UCS of loess, which directly reflects the mechanical properties of the stabilized loess. As shown in Figure 10a,b, the UCS of the stabilized loess increased with the addition of either CCR or PG alone, and the improvement in UCS was greater when CCR was added alone. When the CCR content was 12%, the UCS reached 342.2 kPa, whereas when the PG content was 18%, the UCS was only 107 kPa. With the combined addition of CCR and PG, the UCS of the stabilized loess increased further, indicating a positive combined improvement effect between CCR and PG [7,13]. Compared with the UCS of untreated loess (38.3 kPa), the UCS reached 520 kPa at a CCR content of 4% and a PG content of 18%, representing an increase of approximately 1 257%. This was the highest UCS among all mixtures investigated in this study.

As shown in Figure 10a, when the PG content was 6%, 12%, or 18%, the UCS first increased and then decreased with increasing CCR content, reaching its maximum value at a CCR content of 4%. As the CCR content increased from 0% to 4%, the concentration of free Ca^2+^ increased, promoting ion exchange with Na^+^ and K^+^ adsorbed on the surfaces of clay minerals in the loess, such as montmorillonite and illite. This process effectively reduced the thickness of the electrical double layer around the clay particles, weakened the repulsive forces between particles, and compressed the diffuse layer, thereby promoting the flocculation and agglomeration of soil particles. Consequently, larger aggregates were formed, the cohesion between soil particles was enhanced, and the UCS increased. Meanwhile, CCR provided an alkaline environment for the loess and activated reactive SiO_2_ and Al_2_O_3_ components. The resulting C–S–H and C–A–S–H gels cemented the soil particles and filled the pores, further increasing the UCS [20]. In addition, under alkaline conditions, Ca^2+^ supplied by CCR, SO_4_^2−^ supplied by PG, and dissolved aluminate species derived from clay minerals reacted to form AFt, which contributed to the formation of a framework capable of resisting external loads [7]. When the CCR content increased from 4% to 12%, a large amount of hydration products may have rapidly formed and coated the unreacted aluminosilicate components, thereby hindering further reactions among CCR, PG, and the loess and resulting in a decrease in UCS [19,20].

As shown in Figure 10b, when the CCR content was 0%, 4%, or 8%, the UCS of the stabilized loess increased with increasing PG content. When the CCR content was 12%, however, the UCS first decreased and then increased with increasing PG content.

### 3.3. Water Stability of Stabilized Loess

**Figure 11:**
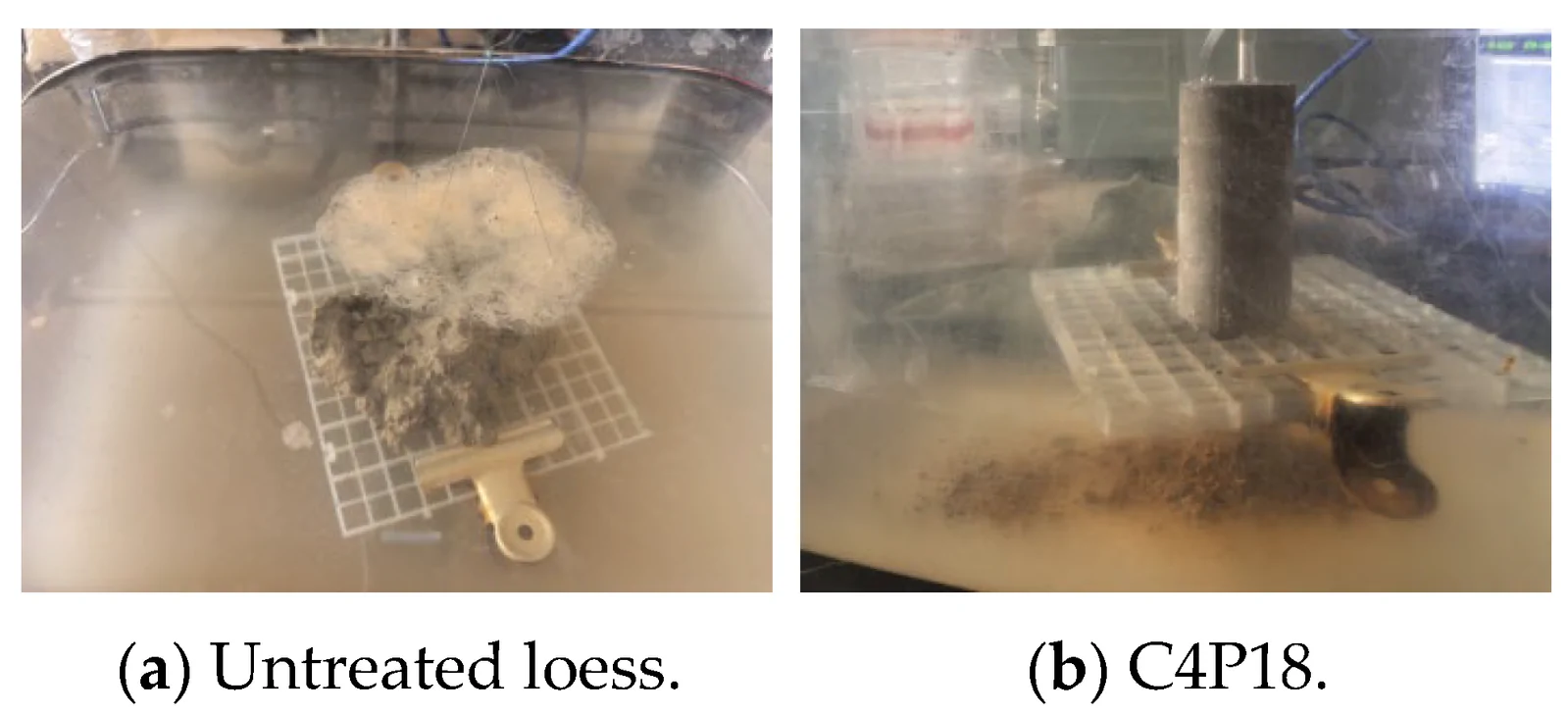
Morphology of soil specimens after water immersion.

The WSC reflects the water stability of stabilized loess and is calculated as the ratio of the UCS after water immersion to that after standard curing. A higher WSC indicates better water stability of the stabilized loess [17,21]. Figure 11 compares the morphological changes in untreated and stabilized loess specimens after water immersion. As shown in Figure 11, the untreated loess specimen disintegrated after immersion, resulting in a WSC of 0, whereas the stabilized loess specimens exhibited no obvious cracking or fragmentation after immersion, indicating improved structural integrity. Table 5 presents the UCS and WSC of the specimens subjected to 27 d of standard curing followed by 1 d of water immersion. As shown in Table 5, the UCS after immersion was lower than that under standard-curing conditions. This reduction may be attributed to the weakening of cohesion between solid particles and deterioration of the pore structure caused by water immersion [17,21].

**Figure 12:**
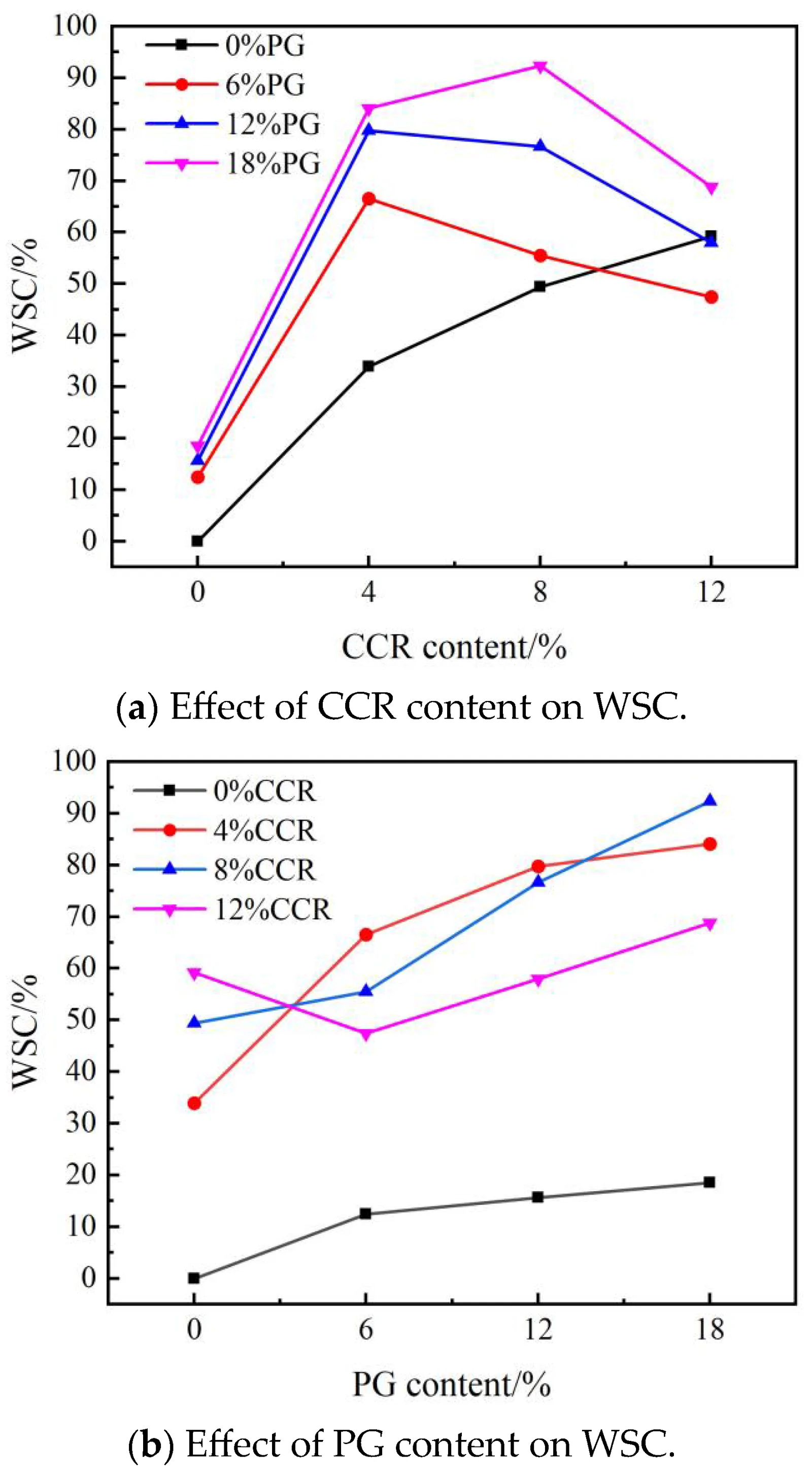
Effect of stabilizer content on WSC.

Based on the UCS values after water immersion and under standard curing conditions at different CCR and PG contents presented in Table 5, the WSC was calculated, and its variation trend is shown in Figure 12. As shown in Figure 12, appropriate additions of both CCR and PG improved the water stability of loess. The variation trend of the WSC was generally consistent with that of the UCS. The highest water stability was obtained at a CCR content of 8% and a PG content of 18%, with the WSC reaching 92.4%.

As shown in Figure 12a, when the PG content was 0%, the WSC gradually increased with increasing CCR content. When the PG content was 6% or 12%, the WSC reached its maximum value at a CCR content of 4%. When the PG content was 18%, the WSC reached its maximum value at a CCR content of 8% and then gradually decreased with further increases in CCR content. As shown in Figure 12b, at a constant CCR content, the WSC generally increased gradually with increasing PG content. However, when the CCR content was 12%, the WSC decreased as the PG content increased from 0% to 6% and then gradually increased as the PG content continued to increase. These results indicate that an appropriate combination of CCR and PG produces a positive combined effect, activating the silicoaluminate oxides in the loess, effectively filling the pores, and hindering water infiltration, thereby improving the water stability of the loess [7,12]. However, when the CCR content is excessively high, a large amount of hydration products forms around the soil particles, hindering further reactions and adversely affecting the improvement in water stability.

Based on the combined UCS and water-stability results, C4P18 exhibited the highest 28-day standard-cured UCS of 520 kPa, with a WSC of 84.1%. The WSC of C4P18 was higher than the 80% reference value specified for solid-waste-stabilized soil mixtures in T/CECS 1542-2024 [22]. In comparison, C8P18 exhibited a slightly lower standard-cured UCS of 487.5 kPa but a higher post-immersion UCS of 450.4 kPa and the highest WSC of 92.4%. It should be noted that the total stabilizer content of C4P18 was 22%, lower than the 26% used in C8P18. Considering that strength is an important mechanical parameter for subgrade fill, while adequate short-term water resistance and a lower stabilizer dosage are also desirable, C4P18 was selected as the preferred mixture under the evaluation criteria adopted in this study. Nevertheless, C8P18 exhibited superior short-term water resistance and may be preferable when water resistance is given a higher priority.

## 4. Microstructural Mechanisms of Stabilized Loess

To elucidate the microstructural mechanisms underlying the stabilization of loess by CCR and PG, four representative groups, namely C0P0, C4P0, C0P18, and C4P18, were selected for SEM and XRD analyses. Among them, C0P0 served as the untreated remolded loess control and was used to characterize the microstructure and mineral composition of the unstabilized loess. C4P0 and C0P18 were selected as the single-additive comparison groups corresponding to the CCR and PG contents in the optimal composite mixture, respectively, to analyze changes in the microstructure and mineral composition of the loess under the individual effects of CCR and PG. C4P18 represented the strength-oriented preferred mixture determined from the macroscopic test results and was used to investigate the formation of cementitious products and the interparticle cementation mechanism under the synergistic action of CCR and PG. By comparing these four groups, the mechanisms responsible for the improved strength and water stability of CCR–PG-stabilized loess were further interpreted at the microstructural level.

### 4.1. XRD

**Figure 13:**
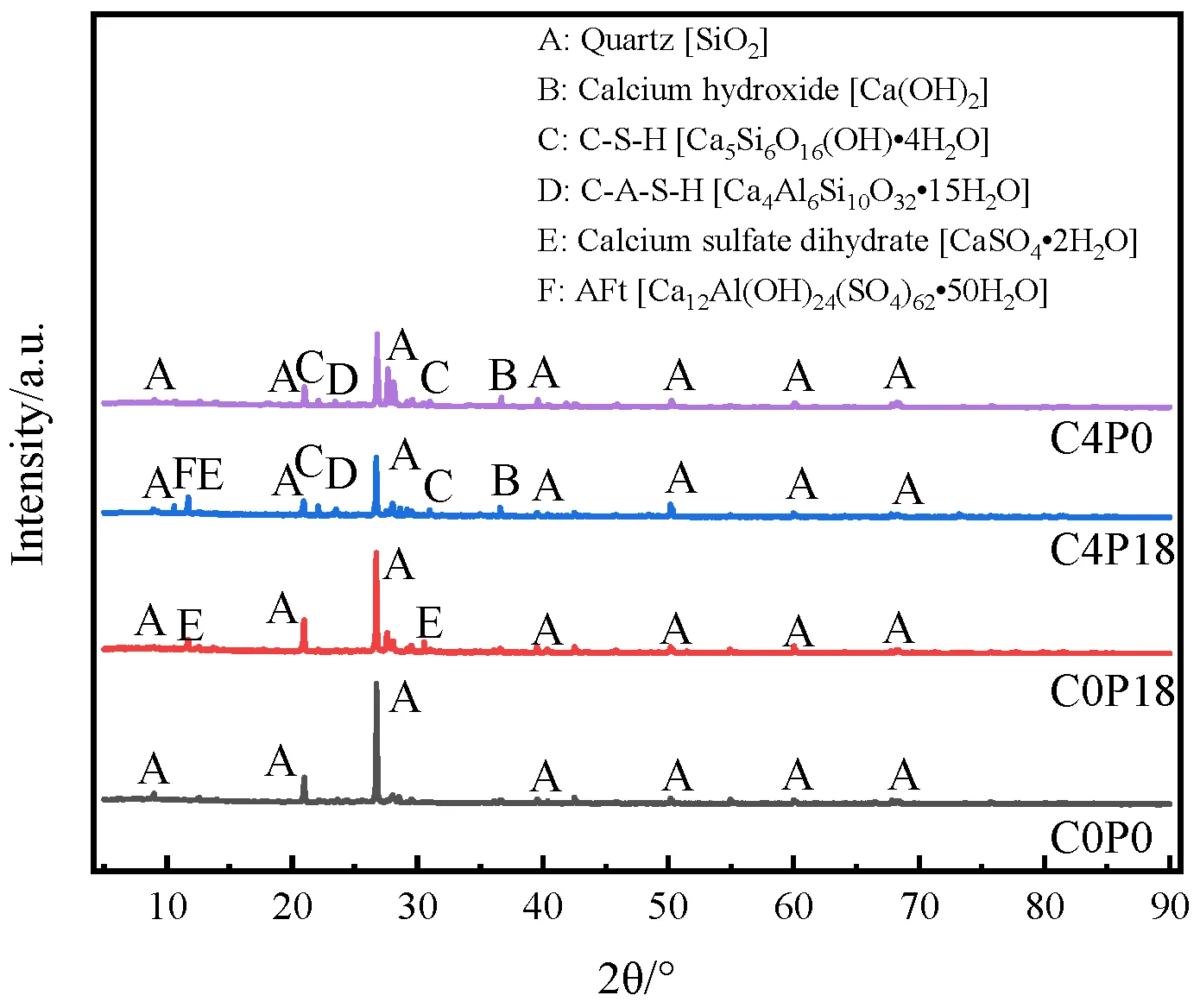
XRD patterns of modified loess under different combination ratios.

As shown in Figure 13, in the single-additive systems, the C4P0 specimen containing only CCR exhibited diffraction features associated with Ca(OH)_2_ and broad features that may be related to poorly crystalline C–S–H/C–A–S–H-type products compared with untreated loess (C0P0), whereas the C0P18 specimen containing only PG exhibited an additional CaSO_4_·2H_2_O diffraction peak. In the composite system, a new AFt phase was observed in the C4P18 specimen compared with C0P0 and the single-additive specimens (C4P0 and C0P18).

Overall, compared with C0P0, the intensity of the SiO_2_ diffraction peak decreased in both the CCR-stabilized specimen and the CCR–PG-stabilized specimen. This may be attributed to the breaking of Si–O and Al–O bonds in the silicoaluminate components of loess under alkaline conditions, resulting in the formation of reactive aluminosilicate species. These species may subsequently react with Ca^2+^ in the system to form poorly crystalline calcium silicate/aluminosilicate hydrate products, which may contribute to the cementation of soil particles and the development of a denser structure [20]. In the composite system, CCR provides an alkaline environment, while PG provides sulfate ions. Their combined action promotes reactions involving the reactive silicoaluminate components in the loess and facilitates the formation of AFt. Together with the possible poorly crystalline calcium silicate/aluminosilicate hydrate products, the formation of AFt may contribute to the development of a denser cemented structure [7,12,23].

### 4.2. SEM

**Figure 14:**
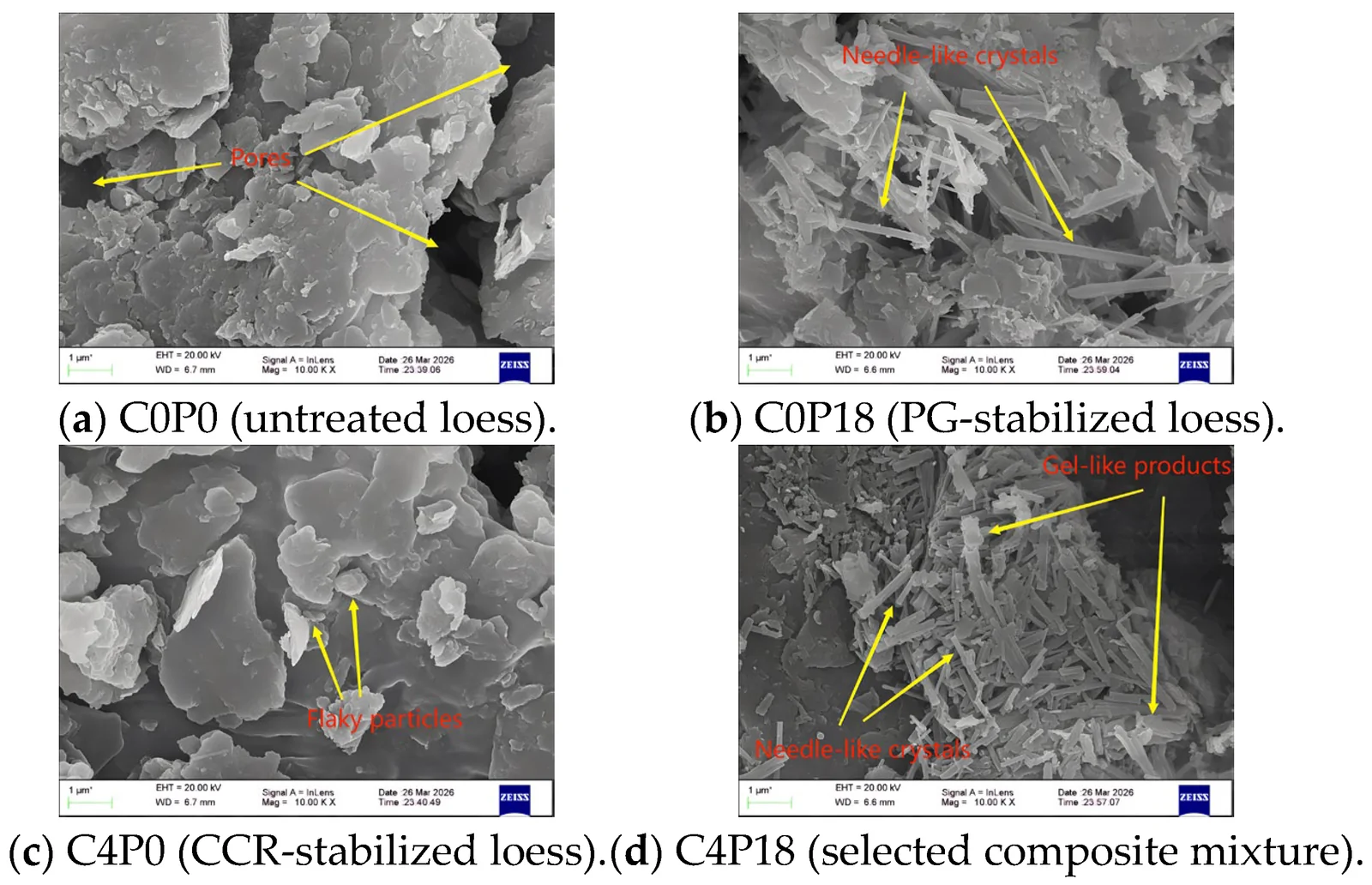
SEM images of stabilized loess with different mixture proportions.

SEM observations of the microstructures of loess stabilized with different mixtures reveal the intrinsic relationship between microstructural characteristics and macroscopic mechanical properties. Figure 14 shows SEM images of untreated loess (C0P0), loess with PG alone (C0P18), loess with CCR alone (C4P0), and loess with the selected composite mixture (C4P18).

As shown in Figure 14a, the untreated loess exhibits well-developed pores and a large number of voids. Particle contacts are mainly point-to-face, resulting in a loose and porous structure. Consequently, the specimen exhibits low UCS and poor water stability at the macroscopic level and is prone to disintegration upon water immersion.

As shown in Figure 14b, when PG is added alone, a small number of needle-like crystals can be observed, providing a certain degree of skeletal support. However, these crystals are distributed relatively independently and fail to form an effective three-dimensional network structure. The relatively high porosity limits the improvement effect, and no substantial increase in UCS or water stability is observed at the macroscopic level.

As shown in Figure 14c, compared with the specimen containing PG alone, the specimen containing CCR alone exhibits better microstructural characteristics. Increased amounts of flake-like and flocculent hydration products fill part of the pores, while enhanced cementation contributes to the formation of a relatively dense skeletal structure. These characteristics correspond to the improvements in UCS and water stability observed at the macroscopic level [18,20].

As shown in Figure 14d, at the optimal composite mixture, the microstructure of the stabilized loess is characterized by the aggregation of small particles into larger particles, accompanied by a change in the contact mode between soil particles, with face-to-face contacts becoming dominant. A large number of dense and interwoven needle-like and prismatic crystals, together with gel-like products, surround the soil particles and cement the originally loose framework into an integrated structure, forming a dense and relatively uniform three-dimensional network. These microstructural characteristics are consistent with the macroscopic test results and support the synergistic stabilization mechanism of CCR and PG in loess [8,23].

### 4.3. Analysis of Microstructural Mechanisms

Based on the macroscopic and microscopic test results described above, the microstructural stabilization mechanism is analyzed as follows. The key mechanism by which CCR and PG jointly stabilize loess lies in the use of their active components to induce the restructuring of the loess mineral framework through the coupled effects of ion exchange, chemical activation, hydration reactions, and physical filling.

When CCR and PG are incorporated into loess, Ca(OH)_2_, the main component of CCR, rapidly dissolves and releases large amounts of Ca^2+^ and OH^−^. This first induces physicochemical modification of the soil particles. Through adsorption and ion exchange, Ca^2+^ replaces low-valence cations such as Na^+^ and K^+^ adsorbed on the surfaces of soil particles, thereby compressing the electrical double layer and reducing the electrostatic repulsion between particles. As a result, the dispersed soil particles undergo flocculation and agglomeration, thereby enhancing the cohesion between soil particles [24,25].

Meanwhile, the high concentration of OH^−^ in the pore solution creates a strongly alkaline environment, which promotes the breaking of Si–O and Al–O bonds in Si- and Al-bearing mineral components of the loess, such as quartz and feldspar, and facilitates the dissolution of reactive SiO_2_ and Al_2_O_3_, thereby providing the necessary silicoaluminate precursors for subsequent hydration reactions. On this basis, the system enters the chemical gelation stage. The dissolved silicate and aluminate species, represented schematically as SiO_4_^4−^ and AlO_4_^5−^, undergo pozzolanic and sulfate reactions with Ca^2+^ and SO_4_^2−^ in the system. SiO_4_^4−^ and AlO_4_^5−^ react with Ca^2+^ to form large amounts of C–S–H and C–A–S–H gels. Meanwhile, SO_4_^2−^ supplied by PG reacts with AlO_4_^5−^ and Ca^2+^ to form needle-like and prismatic AFt crystals [7,12,23].

The C–S–H and C–A–S–H gels coat and cement the soil particles, forming a dense three-dimensional network structure, while the AFt crystals interweave among the soil aggregates to form a high-strength skeleton. These products act synergistically to enhance the cementation strength between soil particles and the overall integrity of the soil structure, effectively inhibiting softening and disintegration caused by water infiltration. Consequently, the engineering properties of the loess are significantly improved, resulting in simultaneous enhancement of strength and water stability [7,12].

## 5. Conclusions

(1) Both CCR and PG can improve the UCS of loess, although their improvement effects differ. When added individually, the UCS of stabilized loess increased with increasing CCR or PG content, and CCR produced a greater improvement in UCS than PG. At a CCR content of 12%, the UCS of loess stabilized with CCR alone reached 342.2 kPa, whereas at a PG content of 18%, the UCS of loess stabilized with PG alone was 107.0 kPa.
(2) The combined addition of CCR and PG exhibited a significant synergistic effect. At a CCR content of 4% and a PG content of 18%, the UCS of the stabilized loess after 28 d of curing reached 520 kPa, representing an increase of approximately 1257% compared with untreated loess. This improvement is attributed to the alkaline environment and Ca^2+^ provided by CCR and the SO_4_^2−^ provided by PG, which may promote reactions involving reactive silicoaluminate components in the loess and the formation of AFt and poorly crystalline calcium silicate/aluminosilicate hydrate products, thereby contributing to the improved structural integrity of the soil.
(3) Among the investigated mixtures, C4P18 exhibited the highest 28-day standard-cured UCS of 520 kPa and a WSC of 84.1%, which was higher than the 80% reference value adopted for solid-waste-stabilized soil mixtures in T/CECS 1542-2024 [22]. C8P18 exhibited the highest WSC of 92.4% and a higher post-immersion UCS than C4P18. However, C4P18 had a lower total stabilizer content (22%) than C8P18 (26%). Considering the high standard-cured UCS, adequate short-term water resistance, and lower stabilizer dosage, C4P18 was selected as the preferred mixture under the evaluation criteria adopted in this study. C8P18 may nevertheless be more advantageous when short-term water resistance is the primary consideration.

This study primarily investigated the effects of different stabilizer contents on the mechanical properties and water stability of stabilized loess under a curing period of 28 d. Since the hydration reactions in the CCR–PG stabilization system continue over time, future research will investigate the evolution of performance under different curing ages, long-term water immersion, and wet–dry cycles to more comprehensively evaluate its long-term service performance.

## Figures and Tables

**Table 1:** Basic physical parameters of loess.

Liquid Limit/%	Plastic Limit/%	Plasticity Index/%	Optimum Moisture Content/%	Maximum Dry Density/g.cm^−3^
26	16.5	9.5	18.0	1.75

**Table 2:** Chemical compositions of CCR.

Raw Materials	Chemical Composition/%						
	CaO	SiO_2_	Al_2_O_3_	MgO	SO_3_	Fe_2_O_3_	Others
CCR	79.89	3.39	2.39	0.17	0.87	0.42	12.87

**Table 3:** Chemical compositions of PG.

Raw Materials	Chemical Composition/%						
	SO_3_	CaO	SiO_2_	Al_2_O_3_	MgO	Fe_2_O_3_	Others
PG	54.38	35.36	8.92	0.64	0.18	0.48	0.04

**Table 4:** Mix proportions of CCR–PG-stabilized loess.

Specimen ID	Mix Proportion (Mass Ratio)	
	CCR/%	PG/%
C0P0	0	0
C0P6	0	6
C0P12	0	12
C0P18	0	18
C4P0	4	0
C4P6	4	6
C4P12	4	12
C4P18	4	18
C8P0	8	0
C8P6	8	6
C8P12	8	12
C8P18	8	18
C12P0	12	0
C12P6	12	6
C12P12	12	12
C12P18	12	18

**Table 5:** Water stability coefficient of stabilized loess.

Specimen ID	UCS After 1 d of Water Immersion/kPa	UCS After Standard Curing/kPa	Water Stability Coefficient/%
C0P0	0	38.3	0
C0P6	9.0	72.2	12.5
C0P12	14.1	90.2	15.6
C0P18	19.8	107.0	18.5
C4P0	66.6	196.1	33.9
C4P6	217.4	326.7	66.5
C4P12	344.8	432.1	79.8
C4P18	437.4	520	84.1
C8P0	141.2	285.6	49.4
C8P6	168.3	303.3	55.5
C8P12	294.9	384.9	76.6
C8P18	450.4	487.5	92.4
C12P0	202.8	342.2	59.2
C12P6	130.2	274.3	47.5
C12P12	194.0	334.8	58.0
C12P18	273.5	397.5	68.8

## Data Availability

The original contributions presented in this study are included in the article. Further inquiries can be directed to the corresponding author.
